# The sterile insect technique is protected from evolution of mate discrimination

**DOI:** 10.7717/peerj.13301

**Published:** 2022-04-18

**Authors:** James J. Bull, Richard Gomulkiewicz

**Affiliations:** 1 Biological Sciences, University of Idaho, Moscow, ID, United States of America; 2 School of Biological Sciences, Washington State University, Pullman, WA, United States of America

**Keywords:** Population suppression, Evolution, Sterile insect technique, Extinction, Female choice, Population dynamics, Mathematical model

## Abstract

**Background:**

The sterile insect technique (SIT) has been used to suppress and even extinguish pest insect populations. The method involves releasing artificially reared insects (usually males) that, when mating with wild individuals, sterilize the broods. If administered on a large enough scale, the sterility can collapse the population. Precedents from other forms of population suppression, especially chemicals, raise the possibility of resistance evolving against the SIT. Here, we consider resistance in the form of evolution of female discrimination to avoid mating with sterile males. Is resistance evolution expected?

**Methods:**

We offer mathematical models to consider the dynamics of this process. Most of our models assume a constant-release protocol, in which the same density of males is released every generation, regardless of wild male density. A few models instead assume proportional release, in which sterile releases are adjusted to be a constant proportion of wild males.

**Results:**

We generally find that the evolution of female discrimination, although favored by selection, will often be too slow to halt population collapse when a constant-release implementation of the SIT is applied appropriately and continually. The accelerating efficacy of sterile males in dominating matings as the population collapses works equally against discriminating females as against non-discriminating females, and rare genes for discrimination are too slow to ascend to prevent the loss of females that discriminate. Even when migration from source populations sustains the treated population, continued application of the SIT can prevent evolution of discrimination. However, periodic premature cessation of the SIT does allow discrimination to evolve. Likewise, use of a ‘proportional-release’ protocol is also prone to escape from extinction if discriminating genotypes exist in the population, even if those genotypes are initially rare. Overall, the SIT is robust against the evolution of mate discrimination provided care is taken to avoid some basic pitfalls. The models here provide insight for designing programs to avoid those pitfalls.

## Introduction

The sterile insect technique (SIT) is used to suppress insect populations by releasing large numbers of individuals (chiefly males) that mate in the wild but sterilize the broods of their mates (Knipling, 1955; Knipling, 1959; Knipling, 1960; Davidson, 1974; Pal, 1974; Klassen & Curtis, 2005; Dyck, Hendrichs & Robinson, 2005; Dyck, Hendrichs & Robinson, 2021). The SIT has many successes and failures over a range of species attempted. Historically, the SIT relied on irradiation or natural incompatibilities to create offspring inviability (Laven, 1967; Vanderplank, 1944), but genetic engineering now affords alternatives (*e.g.*, Alphey, Bonsall & Alphey, 2011; Darrington et al., 2017; Watkinson-Powell & Alphey, 2017; Knudsen et al., 2020; Li et al., 2021), as does the bacterial symbiont *Wolbachia* (Pagendam et al., 2020). The method not only remains widely used but appears poised for an increase in popularity. Its main downside would seem to be husbandry and delivery—the need for infrastructure to artificially rear and release millions of insects.

Population extinction from the SIT is not assured, but certain demographic and ecological properties of the target species are known to increase the odds (Serebrovsky, 1940; Klassen & Curtis, 2005; Itô & Yamamura, 2005; Barclay, 2005; Barclay, 2021). Furthermore, an application may lead to suppression without eradication, in which case sterile males must be released indefinitely. Ongoing use of the SIT raises the possibility of ‘resistance’ evolution, which could be the evolution of genes that block the lethal effect in embryos (Alphey, Bonsall & Alphey, 2011) or, as considered here, wild females that discriminate against sterile males. Resistance evolution plagues many other types of population suppression methods, and it would seem that the SIT is no exception (Richardson, 1979). Yet the SIT has only rarely been reported to experience the evolution of female discrimination (Whitten & Mahon, 2005).

One possibility for the rarity of resistance evolution is that discrimination cannot evolve, because the sterile males are of the same strain as the wild-type males and thus indistinguishable. But SIT males are typically reared in a factory environment where they may be selected to diverge in mating behavior. In some applications, the sterile males are even genetically endowed to be different from wild males (which underlies the basis of sterility, Alphey, Bonsall & Alphey, 2011; Watkinson-Powell & Alphey, 2017). Discrimination would thus seem to be feasible in some systems.

The other possibility—the one explored here—is that selection for discrimination is ineffective, whether at favoring discrimination or in rescuing the population from oblivion despite selection. This possibility is intriguing, as it may suggest other types of interventions that would be protected against evolution of resistance. However, if the SIT is indeed protected against the evolution of discrimination, that protection is likely to be limited to specific contexts that vary with ecology, being affected by such factors as migration, levels of sterile releases, pre-existing discrimination, and many others. Understanding the types of practices that dilute that protection and allow discrimination to evolve should be useful in extending the life of the SIT in any application.

An important distinction separates the evolution of mate discrimination in the wild population from evolution of factory males to become less effective at mating in the wild. Practicality and economics will usually dictate maintaining an ongoing factory population for many generations rather than replacing it often. However, the downside of maintaining a long term stock is adaptation: any factory population will be selected for enhanced growth in the factory environment. Although adaptation to the factory environment should improve production, any factory adaptation of males may render them less fit at mating in the wild—as was observed in the screw worm program (Richardson, 1979). Reduced male mating efficacy has the same demographic effect as evolution of female discrimination, but it is relatively easily corrected by replacing the factory population with wild progenitors. Indeed, factory adaptation led to waning efficacy of the screw worm control program in the 1970s, and replacement of the factory stock with wild stock restored program efficacy (Richardson, 1979). In contrast, evolution of discrimination by wild females may be a permanent impediment to a program.

Here, we consider the evolution and demography of female discrimination under the SIT. Most model details are relegated to an Appendix, and the body of the paper explains the points with a minimum of math. The basics are in fact straightforward.

### SIT basics: population collapse accelerates over time

To set the stage for our study, we provide a brief introduction to some fundamental properties of the basic SIT. The essence of suppression under the SIT is described by a fertility function (*P*, following Barclay, 2005), which gives the probability of a female mating with a wild-type male when sterile males are present:

(1) \begin{eqnarray*}P=\pi M/(\pi M+S).\end{eqnarray*}

S is the density of sterile males, *M* is the density of fertile males, and *π* is female preference for wild-type over sterile males (*π* ≥ 1). Except for the preference, this formula is the same as the numerical model proposed by Knipling (1955) and mathematically equivalent to a fertility function in which males are inferior to wild-type at mating (Barclay, 2005; Barclay, 2021), but using *π* in this way allows the emphasis to be placed on female discrimination.

Whether the population grows or declines not only depends on *P* but also on the birth rate, *b* (the number of daughters born to a female who survive to reproduce). The population will decline if *b P* <1. Birth rates of wild populations are usually considered to obey density dependence, whereby more young are born than will survive to reproduce. Thus *b* itself is not necessarily constant across all conditions. Our numerical analyses (based on the continuous-time model in the appendix) incorporate density dependence, but some of our derivations using discrete-time models omit density dependence (justified on the grounds that, as the population starts declining, density dependence will then become negligible).

**Figure 1:**
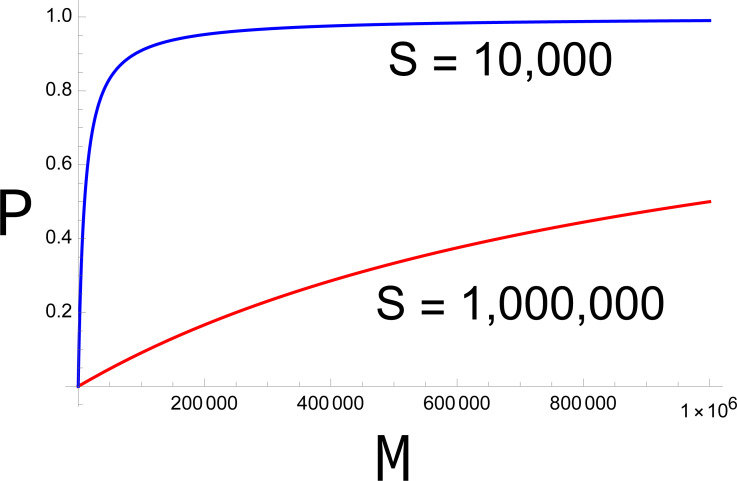
Plots of *P* (probability of mating with a fertile male) across different densities of fertile males (M). In the absence of discrimination (*π* = 1), the blue curve represents *S* = 10,000, the red curve *S* = 1,000,000. The larger release rate (red) has a much stronger effect in suppressing fertility, but even the smaller release has a large effect at small values of M. The curves equally represent other combinations of *π* and S, the red curve representing *S* = 1,000,000*π* and the blue curve representing 10,000*π*. Curves are drawn from Eq. 1.

The fertility function *P* has several interesting properties. First, for a fixed sterile release rate (*S*), *P* is monotonically increasing with *M*, but the increase is decelerating (Fig. 1). Second, *P* approaches 0 as *M* approaches 0. Together, both properties imply that, once the population starts to decline, it continues to decline ever faster. Equally, there is a threshold value of *M* below which any fixed level of preference *π* ceases to rescue the population (at which *b P* <1).

In any dynamic process, the fertility function changes with time because the population density of wild males (and females) is changing. A more accurate formulation of Eq. 1 is thus

(2) \begin{eqnarray*}{P}_{t}=\pi {M}_{t}/(\pi {M}_{t}+S),\end{eqnarray*}

with a subscript *t* indicating time or generation number. (We will assume the release rate *S* remains constant unless indicated otherwise.) This formula is not a recursion, but by assuming the number of adult females equals the number of adult males (*F*_*t*_ = M_*t*_, hence a sex ratio of 1:1), a fixed birth rate of *b* reproductive daughters (and *b* sons) per female such that M_*t*+1_ = b P_*t*_, *F*_*t*_ = bP_*t*_M_*t*_, it follows that, for *π* = 1,

(3a) \begin{eqnarray*}{P}_{t+1}={M}_{t+1}/({M}_{t+1}+S)=b{P}_{t}{M}_{t}/(b{P}_{t}{M}_{t}+S)={P}_{t}/[{P}_{t}+S/(b{M}_{t})]\end{eqnarray*}

and from S / M_*t*_ = (1-P_*t*_)/P_*t*_,

(3b) \begin{eqnarray*}{P}_{t+1}={P}_{t}/[{P}_{t}+(1-{P}_{t})/(b{P}_{t})].\end{eqnarray*}

If *b P*_*t*_ *<1*, then *P*_*t*+1_ will be less than *P*_*t*_. If this inequality is met, the proportional decline in *P*_*t*_ will accelerate with increasing *t*, so there is no stopping. (The formula is trivially modified for an unequal adult sex ratio, but the main points below are unaffected by introducing this complication).

## Materials & Methods

Our results are mathematical and computational, with basic derivations provided in Results. The equations used for all except Fig. 1 are provided in an Appendix. The code to generate the figures are provided as Mathematica files.

## Results

### When rare, discrimination need not rescue a collapsing population

**Figure 2:**
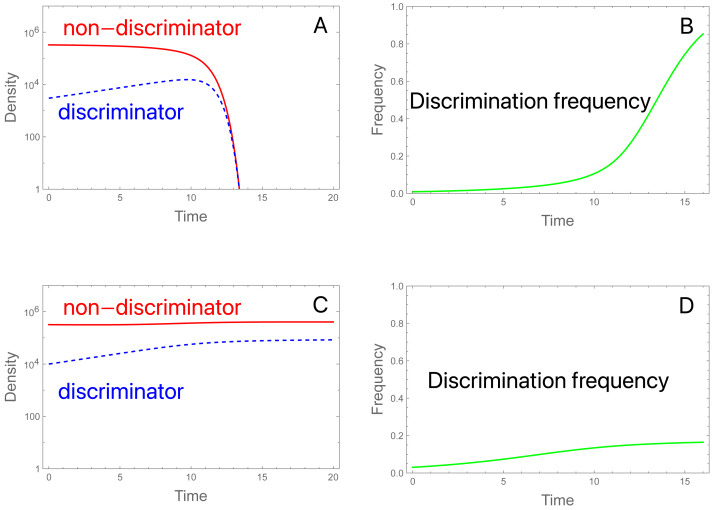
Initial frequencies determine collapse or persistence of a population in the presence of discriminating females that could in principle rescue the population. A and C display females of each type over time (or equally of males); B and D display frequencies of discrimination. Total initial density of females (and of males) is 3.3 × 10^5^ in both trials, but the fraction of discriminators differs. (A) Initial discrimination density is not sufficient to rescue. The density of discriminating females (blue, dashed) increases initially while the density of non-discriminating females (red) declines throughout. As the non-discriminator density continues to fall, even discriminating females become overwhelmed by steriles and eventually decline. Initial frequency of discriminators is 0.91%. (B) Evolution corresponding to (A): the frequency of discriminating females increases throughout the process even as the population has crashed. (C) Initial discrimination density is sufficient to rescue. The only change from (A) is that the initial frequency of discriminators is increased to 3.03%. (D) Evolution of discrimination is rapid until the population has recovered but continues more slowly afterward. Evolution slows because the population is at carrying capacity, hence the birth rate (and potential for evolution) is determined by the intrinsic death rate; discriminators eventually displace the wild-type. It is noteworthy that evolution is faster in the population going extinct. Parameters are *S* = 3 ×10^6^, *b* = 10, *K* = 10^6^, *π* = 4.0, *δ* = 0.01; see the Appendix for the model equations. Note that the density of males of each genotype equals the density of females of each genotype. Although not illustrated, increasing *S* to 3.15 × 10^6^ in (C) results in extinction, so a slight increase in sterile release overcomes the failure cause by discrimination.

The properties of *P*_*t*_ provide insight to the evolution of female preference, and as will be shown, evolution is unlikely to rescue a population that is collapsing under the SIT when discrimination is initially rare (Fig. 2). Suppose that most of the females lack preference (*π* = 1) but a small fraction (denoted by *) has a potentially strong preference for fertile males. At the start (*t* =0), the fertility function for each type of female is

(4) \begin{eqnarray*}{P}_{0}={M}_{0}/({M}_{0}+S)~~~~\text{(no-preference females)}.{P}_{0}^{\ast }=\pi {M}_{0}/(\pi {M}_{0}+S)~~~~\text{(discriminating females)}.\end{eqnarray*}

We will consider the favorable case for discrimination that *b P*_0_<1 but *b P*_0_^*^ >1, *i.e.,* that the SIT will cause an initial decline in a population of mostly non-discriminating females, but preference is strong enough to rescue a population of the current size if all females possess it.

The key to this process, and the reason that a low frequency of discriminating females will not escape extinction, is that the same pool of wild-type males is common to both fertility functions—*M*_*t*_ is the same for both types of females (assuming the genetic basis of the female preference does not affect male fitness). With initiation of the SIT, the total population declines because most females have no preference and, for them, *b P*_0_<1. Under a fixed progeny sex ratio (*e.g.*, 50% sons), this leads to a *progressive* decrease in the population size/density of fertile males: *M*_0_ >*M*_1_ >*M*_2_ >*M*_3_ and so on, with concomitant effects on the fertility function *P*_*t*_.

Even when discrimination increases initially, its long term persistence is not assured (Fig. 2). Indeed, the total population will decline even though discriminating females increase in density at first. But as M_*t*_ drops, it can eventually descend to a level at which *b P*_t_^*^ <1; henceforth, discriminating females are also declining in numbers but not frequency. If discriminating females comprise enough of the population, or if the release rate *S* is small enough, then the early increase of discriminating females may halt the population decline. But when discriminating females start out rare, their density increases too slowly to reverse the decline.

The preceding addresses population sizes, not evolution. As long as sterile males are released, the discrimination allele (*A*) always increases in frequency (although the universality of this outcome requires an intrinsic death rate greater than 0 in the model). Despite the increase in the frequency of *A,* the discrimination allele, the absolute abundance of discriminating females avoids decline only if the total fertile male population is sufficient to maintain adequate fertility. The process is a race between adaptive evolution (with discrimination continuing to evolve) and population decline, much as in other evolutionary rescue problems (elaborated below). One difference from standard evolutionary rescue is that the decline in numbers of discriminator females is affected by the background abundance of non-discriminators. Persistence is enhanced by fertile males of either type, allowing females to avoid sterile matings. Thus rescue in this case depends on the entire population, not just the subset of individuals with the best genotypes. However, the effect is not strictly frequency-dependent selection (Svensson & Connallon, 2019).

Figure 2 offers a minimal sample of parameter values and initial conditions. One outcome not necessarily evident from the figure is a dependence of evolution and dynamics on initial densities. When the population is near carrying capacity, evolution is slow (and sterile releases are less effective) because there is little opportunity for growth: the model multiplies log fecundity by (1 − *N*/*K*), and as this density-dependent term approaches zero, there is little addition to the population. Thus, fitness differences due to discrimination vanish and even sterile releases have little effect on evolution or population density. This works against preference evolving fast enough to sustain a declining population, but it is offset by the intrinsic death rate (artificial population suppression, as with an insecticide, would have a similar effect). Our trials thus started away from carrying capacity and assumed a modest intrinsic death rate. A different model of density dependence might lack this property.

The criterion *b P*_0_^*^ >1 requires that discrimination (*π*) must be sufficiently strong for the abundance of discriminating females to increase initially. How strong? Inserting the expression for *P*_0_^*^ and rearranging shows that *π* ≥ *π*_min_*M*_0_) [*S/(b − 1)* ], where *π*_min_ is the minimum discrimination required for preference to increase initially. (*π*_min_ is equally the minimum population-wide preference required to prevent population decline at the onset of release rate *S*.) *π*_min_ increases with *S* and decreases with *b* and *M*_0_. Since the absence of preference in non-discriminating females is represented in the model by *π* = 1, the minimum effect of a mutation that would enable rescue is *π*_min_ –1. (Note that this result merely implies that a preference level of *π*_min_ would begin to increase at the onset of sterile release; it does not ensure that the increase will continue as the population declines—see above). This suggests that rescue will require relatively large-effect mutations when employing large sterile releases (*S*) or when non-discriminator birth rates *b* are close to 1 (*i.e.,* just above replacement). And, to the extent that mutations of large effect are rare, the conditions for SIT resistance to arise *de novo* should also be rare.

The joint population and evolutionary dynamics of resistance to SIT are similar to those of “black-hole sink” adaptation (Holt & Gaines, 1992). A black-hole sink is a population that is a demographic sink but receives a recurrent stream of maladaptive immigrants that maintain a local population at low abundance. In the original concept, cutting off immigrants would doom the population because, as a demographic sink, it will by definition go extinct when isolated (Pulliam, 1988). In the SIT version, however, it is the introduced sterile males that doom the population; extinction would be avoided were the steady flow of steriles interrupted. In black-hole sink models, the evolution of genotypes that would save the population from extinction with or without the immigrant stream is predicted to be rare (Holt & Gomulkiewicz, 1997; Gomulkiewicz, Holt & Barfield, 1999; Holt, Gomulkiewicz & Barfield, 2003). This is used to help explain “niche conservatism”—which is the observation that species only rarely adapt to conditions outside their fundamental niche (Holt & Gaines, 1992). Our argument here for the rarity of resistance evolution to SIT is similar and presumably should extend to systems that assume more complex genetic bases for resistance.

### Proportional releases are prone to escape extinction

An alternative to constant-*S* release is a proportional release, as was considered in models for the evolution of resistance to a version of the SIT using a dominant embryonic lethal (Alphey, Bonsall & Alphey, 2011; Watkinson-Powell & Alphey, 2017). A proportional release means that *S* is now varied throughout the intervention, with *S*_*t*_ being chosen each generation to maintain the same value of the fertility function across time:

*P*_*t*_ = *M*_*t*_/(*M*_*t*_ + *S*_*t*_) = 1/(1 + *S*_*t*_/*M*_*t*_) = 1/(1 + *d*), 

where *M*_*t*_ is the combined density of all males. (The notation *S*_*t*_*/M*_*t*_ =* d*, where *d* is a constant, is from Alphey, Bonsall & Alphey (2011)). The constancy of the ratio *S*_*t*_/ *M*_*t*_ means that not only the discriminator fertility function *P* is constant throughout the intervention, but so is the fertility function for discriminators (*P*^∗^ in Eq. (4)).

Intuitively, this strategy seems as if it must be less effective than a constant-*S* release of the same initial impact, as it lacks the accelerating efficacy of steriles as the population declines. But any intuition that it is less effective leaves open many possibilities –that constant-*S* release may still be an effective intervention, and of course, it is an intervention that demands less effort. In any actual implementation, maintaining a constant proportion of steriles would be at best approximate, but for the sake of modeling, we assume it is perfect.

To illustrate the contrast between a proportional-*S* protocol and a constant-*S* protocol, we again consider the case that *b P*_0_<1 but *b P*_0_^*^ >1. One difference between the two protocols is immediately evident: as the values of *P* and *P*^∗^ do not change throughout treatment, the fact that *b P*_0_^*^ >1 should mean that discrimination is never lost no matter what its initial frequency –there should be no effect of a high abundance of non-discriminators overwhelming discriminators. If true, that would be a major difference from constant-*S* releases: the long term outcome of a proportional-*S* release is not affected by the initial abundance of discriminators. This intuition proves to be correct in some respects and false in others.

**Figure 3:**
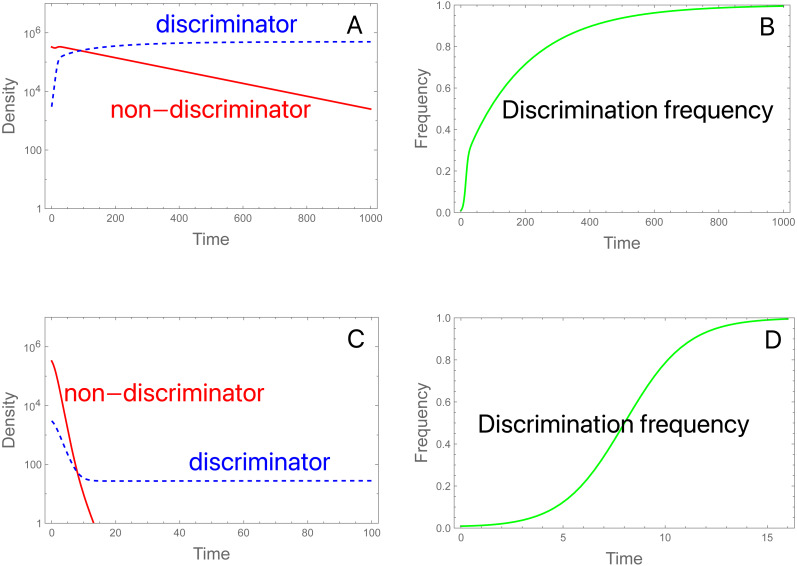
Population dynamics and evolution of discrimination with proportional releases of steriles. (A, B) Initial conditions and parameter values are the same as in Fig. 2A, but here the population persists. As before, evolution favors discriminators. *bP* = 0.99, and bP^*^ = 3.056. (C, D) The same initial conditions are used as in (A, B) except that *S* is increased from 3 × 10^6^ to 11.754 × 10^6^, which reduces the fertilities. Now there is an initial rapid decline in population size, but discriminators ultimately persist and grow slowly. *bP* = 0.27, and bP^*^ = 1.0103. These trials assigned an intrinsic death of 0.01 to all genotypes, so fertilities should subtract the deaths, but once adjusted, the two cases satisfy net fertility < 1 for non-discriminators and *net fertility* > 1 for discriminators. Note the different time scales—evolution is much faster in (D) than in (B).

Two proportional-*S* trials are shown in Fig. 3. Both satisfy *b P* <1 and b P^*^ >1. Figures 3A and 3B use the same initial conditions and parameter values as in Figs. 2A, 2B; for those values, *b P* is just under 1 and b P* exceeds 3. With constant-*S*, the population was extinguished; under proportional-*S*, the population never falls below 10^5^, and the *A* allele gradually displaces allele *a*. Figures 3C and 3D use the same inputs except that the initial *S* is raised from 3 ×10^6^ to 11.7 ×10^6^. Now, the initial population falls rapidly, but discriminators are ultimately maintained at a low (but gradually increasing) level. Thus, the expectation is met in both cases that b P^*^ >1 ensures maintenance of discriminators. An obvious concern with proportional releases is suggested by Fig. 3C: population suppression works initially against all genotypes, but it ceases to become effective when non-discriminators are purged. There would likely be no way at the outset of an intervention to anticipate the presence of rare discriminators that were capable of avoiding eradication. Of course, and as before, increasing the release rate sufficiently would work to eradicate because a sufficiently higher release rate will drive b P*<1.

**Figure 4:**
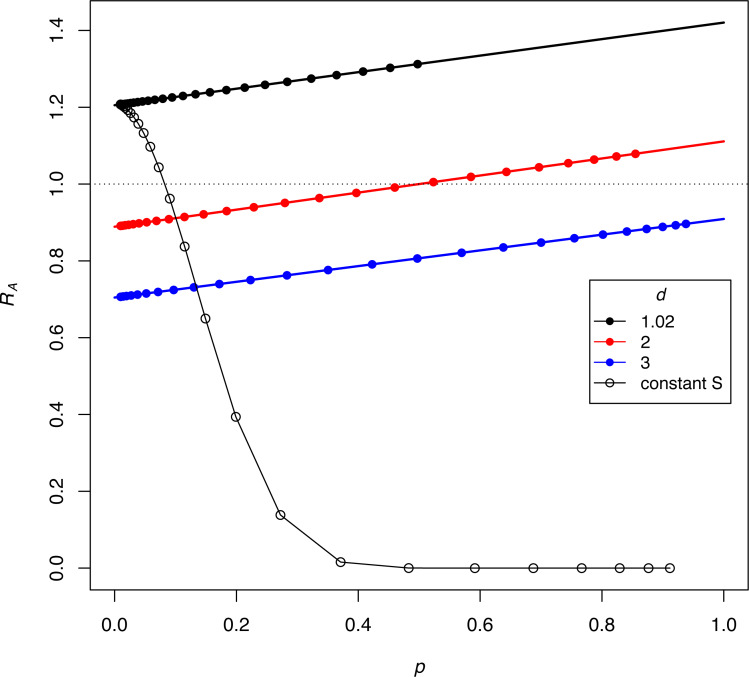
Interaction between the frequency and growth rate of discriminators for constant-proportion implementations of the SIT (the three lines with closed symbols) and one implementation under constant-*S* (the black curve with open symbols). Release rates are given as *d* =* S/M*, as per the key. The vertical axis (*R*_*A*_) is the per-generation, absolute finite growth rate of discriminators, a value exceeding 1.0 meaning that their density increases. Dots are placed at the successively increasing values of *p* as the population evolves for 25 generations, starting with *p* = 0.91% discriminators. Constant-proportion SIT is shown with the 3 lines: green, red, and black. The numerical increase or decrease of discriminators depends on their frequency (*p*), although the black line has discriminators increasing (*R*_*A*_ > 1) at all values of *p*, and the green line never experiences growth (*R*_*A*_ < 1). For the red line, discriminators decrease in density while their frequency is ‘low’ but eventually increase once their frequencies attain a sufficient value. The black (declining) curve depicts the process for a constant-*S* implementation under the same initial conditions as the black line, and a dramatic difference is easily seen. For all trials, *π* = 2.5, *b* = 2.0, thus *bP*^∗^ = 1.42, 1.11, and 0.91 for the black, red, and green lines, respectively. As shown for the blue line (for which bP^*^ < 1), the population never experiences *R*_*A*_ = 1, hence discriminators are lost. The model assumes discrete generations (appendix).

The dynamics in (3C, 3D) reveal the anomalous result that females of genotype *A* decrease in absolute density until genotype *a* is largely purged, with allele *A* henceforth maintaining an almost constant density (as its b P* barely exceeds 1.0, growth is expected to be minimal). If b P* exceeds 1, why does the density of discriminators decrease initially? This behavior appears to reflect a ‘poisoning’ from non-discriminators. Inspection of the equations indicates that the initial drop in the absolute abundance of *A* (despite its monotonic increase in frequency) stems from sexual reproduction. Mating of *A* females with *a* males (which is essentially a loss in fecundity for *A*) is not returned in magnitude when *A* males mate with the lower-fecundity *a* females to produce *A* progeny. Derivations (appendix, assuming discrete time) show the interesting effect that the density of discriminators increases or decreases according to their frequency –an interaction between genetic evolution and population growth. Figure 4 provides numerical examples: for different ratios of *S/M* (= *d*, in the figure), the finite growth rate of discriminators (*R*_*A*_) starts above or below 1 at low frequencies of discriminators and rises above 1 at high frequencies. The lines lie entirely below *R*_*A*_ =1 if b P*<1.

A similar process must occur with constant-*S* releases but is not easily derived in such a clear fashion as for proportional-*S* releases. For one case, Fig. 4 therefore also plots the dynamics of constant-*S* release for comparison to the same initial conditions under proportional-*S*. The difference is profound and shows how vastly more effective a constant-*S* release can be. We return to constant-*S* releases for the remainder of the paper.

### Imperfect SIT and the potential for evolution of discrimination

The previous results showed that extinction in the presence of low initial levels of discrimination is sensitive to the sterile release rate, magnitude of discrimination, and initial abundances. These models assumed a single population with constant release rate, *S*. In practice, the SIT will be applied in a spatial context and perhaps be discontinued when it appears that the population has been eradicated. What is expected when we violate the model to include these more realistic properties? We consider two cases below.

#### Periodic cessation of sterile release without eradication allows resistance to evolve

**Figure 5:**
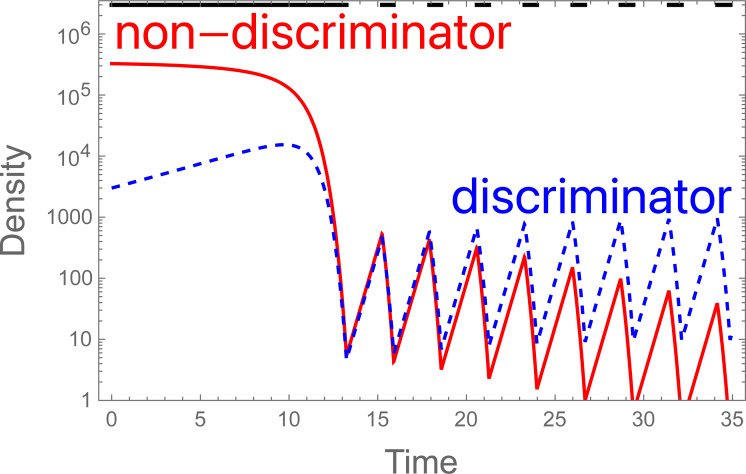
Evolution of discrimination with periodic cessation of the SIT. The jagged, dashed blue is the density of discriminating females, which ultimately ascends, solid red is the non-discriminating female. The horizontal, intermittent black line at the top is the level of sterile males released (*S* in the model, 3 × 10^6^), which is applied whenever the female population exceeds 1,000 and existing sterile male density is no more than 200; sterile release is halted when the female density drops to 10 or less. After attaining the initial suppression by time 13, the SIT continues to suppress whenever applied, just not enough to eradicate because of its cessation whenever the population drops below the threshold. If continuous sterile releases are initiated after time 27 (for example), the population then collapses. Parameter values are as in Fig. 2; initial conditions are as in Fig. 2A. The model is the continuous-time model in the Appendix except for the conditional cessation in *S*.

If releases are relaxed before eradication, then re-implemented as the population rebounds, discrimination will evolve (Fig. 5). Assuming that the population never goes extinct, this evolution of preference has the potential to allow eventual population recovery even during times of sterile release. Suppression would then require increasing the release rate. Even if the release rate, when applied, is sufficient to suppress the population, the evolved preference may spread or seed the evolution of further levels of preference. This result is consistent with analyses of black-hole sink models which show that temporal variation in the sink facilitates adaptative evolution due to the periods of mild demographic conditions (Holt, Barfield & Gomulkiewicz, 2004).

#### Simple cases of migration block evolution

Migration and dispersal present possible reasons that the SIT applied to a specific geographic area will fail to eradicate –despite total suppression of a local population, the target species will be continually re-introduced. The question here is whether those reintroductions facilitate the evolution of female preference. There are countless migration models to consider when varying initial population sizes, compositions, migration rates, and sterile release rates (*e.g.*, Watkinson-Powell & Alphey, 2017 offer one of many alternatives). Generalities may exist in the form of multi-dimensional sets of parameter values that lead to common outcomes, but even those generalities will likely be specific to the nature of the model (*e.g.*, the number of islands, relative island sizes, generations discrete or continuous, which sexes migrate, and migration rates varying through time). Our goal here is merely to consider a few simple cases to illustrate a range of possible outcomes.

A common type of migration model assumes an island population with recurring migration from a mainland population of fixed allele frequency (Crow & Kimura, 1970). Here we assume that the mainland and island initially have the same low frequency of discriminating males and females (0.0091), and migration continually reseeds the island. What evolution is expected in the island population?

**Figure 6:**
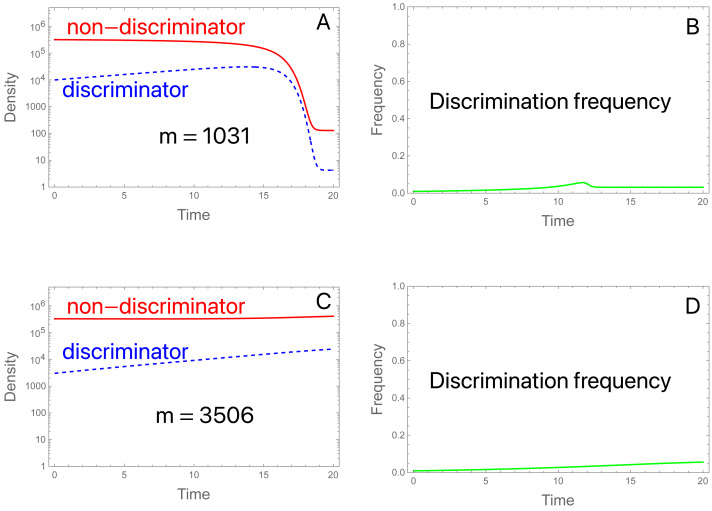
The SIT with migration. (A) Discriminators (dashed blue) are too rare in the initial population and in migrants (at 0. 91%) to prevent suppression, given their preference level (*π* = 2.0). Total migrants are 1,031.25 with 0.91% discriminators in the migrants and in the initial population. The population collapses, whence both types are maintained only through migration, and discriminators are maintained at low frequency (0.032, observed out to 2,000 generations). (B) The density of migrants is slightly more than tripled to 3,506.25, still a small fraction of the initial population (1.1%) and now the population avoids suppression. Although the frequency of discriminators never rises to high levels, population suppression would be achieved in the absence of discrimination. Perhaps surprisingly, the frequency of discriminators equilibrates to 0.043, a value only slightly higher than that in (A, B). Values not given above are *b* = 10, *K* = 10^6^, *S* = 3 × 10^6^; initial conditions are discriminator density (3 × 10^3^), non-discriminator density (3.27 × 10^5^).

We consider two examples with low migration rates. In both, the SIT implementation suppresses the population in the absence of migration. (Aside from migration and reducing *π* to *2*, the initial conditions and parameter values are the same as used in Fig. 2A). The two examples differ only in the level of migrants. (A, B): With migrants at 1,031 per generation and a starting population of 3.3 ×10^5^, the population is suppressed (Fig. 6A). There is a brief and shallow rise in the frequency of discriminators that is squelched as the population crashes and becomes dominated by migrants (Fig. 6B). After collapse, the island population is effectively one in which sterile males are released at a high enough rate to prevent ongoing reproduction by the migrants. Even discriminators cannot reproduce at a high enough level to offset their decline, so they cannot evolve fast enough to offset the influx of migrants dominated by non-discriminators. (C, D): An approximate tripling of migrants radically changes the outcome (Figs. 6C, 6D). The population avoids a crash. Even so, the frequency of discriminators never attains a high level and appears to equilibrate only modestly above its frequency in migrants, even though discriminators are essential to avoidance of population suppression. (If *π* is set to 1 for the trial, the population is suppressed by the SIT).

These two examples merely serve to foreshadow the complexity of the SIT with migration. Initial suppression can be followed by continued suppression despite ongoing migration with discriminators. More surprisingly even when the initial population is not suppressed due to a combination of migration and discriminators, ongoing migration can maintain discriminators at low frequency in the large population. The sensitivity of these outcomes to model structure and parameter values is unknown. Considerable effort may be required to identify generalities that can be translated into robust empirical outcomes.

## Discussion

The sterile insect technique has been used for approximately three quarters of a century (Knipling, 1955; Pal & Whitten, 1974; Dyck, Hendrichs & Robinson, 2005; Dyck, Hendrichs & Robinson, 2021). It is far from universally successful, but its reasons for failure mostly appear to lie in basic demographic realms rather than in the evolution of discrimination by females to avoid sterile males (Klassen & Curtis, 2005; Pal & Whitten, 1974; Davidson, 1974), although discrimination has been reported (McInnis, Lance & Jackson, 1996). It must be acknowledged that female discrimination may be difficult to detect when other reasons for failure are also present, but perhaps the most relevant data are those in which the SIT succeeds and thus does not fall victim to rapid evolution of discrimination.

At one level, it is perhaps a subtle distinction between female discrimination *via* her recognition of sterile males *versus* male incompetence in mating due to either a debilitating method of sterility (*e.g.*, radiation) or their adaptation to laboratory conditions. The key difference between the two processes lies in whether the wild population evolves. Factory adaptation was the basis for a temporary rebound of the U.S. screw worm population, ultimately resolved by replacing the factory population with wild stock (Richardson, 1979). In contrast, if the wild population evolves (to discriminate), replacement of the factory strain will not erase that evolution, although a new factory strain could perhaps be chosen to lack the characteristics that evolved females use to recognize sterile males.

Otherwise, the qualitative results here may be relevant to many if not all the diverse systems used to enforce the sterile insect technique, and even to other forms of resistance (Alphey, Bonsall & Alphey, 2011; Pagendam et al., 2020). However, the problem is complex enough to allow such different modeling approaches as to make comparisons (and thus generalities) difficult. For example, Alphey, Bonsall & Alphey (2011) considered the evolution of resistance to a dominant-lethal SIT implementation under proportional-release. Their parameter-rich model invoked a lot of genetic detail, with most emphasis on genetic equilibria, less on population suppression. Some of their key findings were that evolution of resistance alleles allowed the introgression of the dominant-lethal gene into the target population, but that continued introduction of sterilizing males (which were homozygous sensitive at the resistance locus) could prevent fixation of the resistance allele. In contrast, our approach addressed evolution of female male mating preference under a constant-release implementation, it simplified the genetics to a bare minimum, and it focused on extinction. However, our results for migration did parallel their work in showing that resistance evolution can be suppressed by introgression of non-resistance alleles. Likewise, Watkinson-Powell & Alphey (2017) studied a model of migration in the same framework used by Alphey, Bonsall & Alphey (2011), but, in further contrast to our migration models, considered the (realistic) process of reciprocal exchanges between a suppressed and a non-suppressed population. The bi-directional exchange allows evolution in the treated population to affect the ‘source’ population, which was not allowed in our model. There is thus a wide realm of realistic biology to consider in the evolution of resistance to the SIT, and a robust understanding is likely to require a blend of simple and detailed models. Perhaps the biggest effect to have emerged from our analysis, and one that separates ours from prior work, is the profound difference between releasing a constant density of sterile males *versus* maintaining a constant proportion of sterile males. The proportional-release strategy allows the evolution of even rare discrimination alleles in the population that a constant-release strategy can suppress. Of course, it remains feasible to suppress evolved populations by increasing the release rate under either release strategy.

Our basic result here is that the same dynamic principle that underlies success of the SIT –the accelerating efficacy of brood sterilization as the wild population declines –also underlies the expected failure of evolution of female discrimination against sterile males. The frequency of discrimination does indeed increase from the presence of sterile males. But if discrimination starts rare, it cannot be maintained demographically as the pool of available males shifts increasingly toward 100% steriles. Even if a population that consisted entirely of discriminating females could withstand the demographic impact of sterile releases, rarity of discrimination allows population collapse so that discriminating females increasingly mate with steriles.

Key to this result is that female preference is not absolute. Rather, females of any preference increasingly mate with sterile males as sterile males become proportionally more common, just less so the greater the discrimination. (An absolute preference would be represented as *π* = ∞ in our model.) Empirically, mate preferences are indeed graded within species, though not necessarily so between species (Jennions & Petrie, 1997; Bonduriansky, 2001; Mendelson & Safran, 2021). An absolute preference for wild-type males would be favored and would not face the demographic collapse problem experienced by graded preferences, but there is no obvious mechanism by which an absolute preference could evolve prior to the onset of sterile releases.

In contrast, it seems perhaps plausible that large effect mutations might arise against embryonic lethality: the lethal agent might be blocked by deletions of a key receptor, for example. In this respect, the feasibility of different types of resistance evolution may depend on mutational availability more than the mode of selection.

The nature of female preference, and especially its pre-existence, will likely have a major effect on the outcome of any evolution of discrimination against sterile males. Although at the outset of a SIT implementation there may be no history of selection for females to avoid sterile males, there may have been considerable prior history of mate selection for other reasons, such as to avoid mating with the wrong species or strain (Jennions & Petrie, 1997; *e.g.*, Carlson, Kelly & Couzin, 2020; Carlson, Kelly & Couzin, 2020; Gatto, Loukola & Agrillo, 2022). Such pre-existing strain differences could be a basis for rapid evolution of discrimination against sterile males. Even so, strain differences were a concern with the wide-spread implementation of the screw worm program, yet the program nonetheless did not fall victim to evolution of resistance (Richardson, 1979). The success of the screw worm program may attest to the robustness of suppression by constant sterile releases and easy increases in the release rates when needed. In this sense, the SIT may be a good candidate for management through dynamic programming (Hackett & Bonsall, 2019).

The greatest potential for evolution of mate discrimination under the SIT appears to result from an imperfectly applied SIT, or to the use of a proportional-release strategy. If a portion of the population is allowed to persist amid ongoing (perhaps periodic) constant releases, discrimination will increase in frequency and may eventually reach a level at which control is no longer possible without increasing the sterile release rate. But one should not equate the mere persistence of the population with the evolution of resistance –the wild individuals could be migrants, which do not necessarily pose a threat from evolution of discrimination.

Our results resemble those from studies of evolutionary rescue and adaptation in demographic sinks. That literature (cited above) emphasizes that adverse demography creates obstacles for adaptative evolution even allowing extinction per se (Gomulkiewicz, Krone & Remien, 2017). Likewise, the basic requirement that demographic persistence is necessary (but not sufficient) for adaptive evolution creates an absolute threshold that adaptive genotypes must surmount, with harsher conditions generating ever more imposing genetic barriers. Adaptation in a sink maintained by immigration can be swamped by the arrival of maladaptive genotypes unless the local frequency of adaptive genotypes is sufficiently high. All three kinds of demographic obstacles are substantial and have been advanced as explanations of “niche conservatism”, the observation that species rarely adapt to environments outside of those in which their populations are self-sustaining (Holt & Gaines, 1992). In the context of SIT, this literature, like our results, suggests that the conditions necessary for resistance are stringent and explains why the SIT should be protected from being undermined by the evolution of mate discrimination.

## Conclusions

The question investigated here is whether the sterile insect technique (SIT)–inundating natural populations with individuals of the same species whose matings will sterilize broods–is prone to select wild females that discriminate against mating with the sterilizing males. Our focus was the release of sterile males (not females), and our methods were mathematical. Discrimination constitutes a form of resistance, and by parallel with chemical pesticides and herbicides that are known to lead to the evolution of resistance, the SIT might be thought to face the same problem. Although natural selection does indeed favor discrimination whenever sterile males are released, if the initial application of the SIT is sufficient to initiate population collapse, then any rare genotypes capable of discrimination are not likely to be common enough to offset the population collapse. With the continued release of sterile males and population decline, the probability of mating with a sterile male increases to the point that discriminating females also decline. Migration of wild individuals into a successfully treated zone does not appear to allow discrimination to evolve, but avoiding the evolution of discrimination does require continual sterile release until eradication. However, these results may be specific to constant-release programs, as proportional-release appears to allow for much easier evolution of resistance.

##  Supplemental Information

10.7717/peerj.13301/supp-1 Supplemental Information 1 Code for Fig. 1

10.7717/peerj.13301/supp-2 Supplemental Information 2 Code for Fig. 2

10.7717/peerj.13301/supp-3 Supplemental Information 3 Code for Fig. 3

10.7717/peerj.13301/supp-4 Supplemental Information 4 Code for Fig. 4

10.7717/peerj.13301/supp-5 Supplemental Information 5 Code for Fig. 5

10.7717/peerj.13301/supp-6 Supplemental Information 6 Code for Fig. 6

10.7717/peerj.13301/supp-7 Supplemental Information 7 Appendix of equations used for the figures

## Additional Information and Declarations

### Data Availability

The following information was supplied regarding data availability: Mathematica files, an R Markdown file, and an appendix of equations are available in the Supplemental Files.
