# Supplementary material for: The sterile insect technique is protected from evolution of mate discrimination
Source: PeerJ. 2022 Apr 18;10:e13301. doi: 10.7717/peerj.13301 (PMC9022645; doi:10.7717/peerj.13301)
Supplement: Supplemental Information 7 [file peerj-10-13301-s007.pdf]

Appendix: Formal equations for resistance evolution under the sterile insect technique

Continuous time equations

This appendix establishes the basis of a numerical model that combines evolution of female discrimination with population dynamics. We use a standard logistic model

$$\dot{F} = rF\left(1 - \frac{T}{K}\right) \tag{A1}$$

to represent changes in population sizes, where F is numbers of females (which will be subscripted according to genotype), K is the carrying capacity, and T is the total population size of all individuals (males and females). Under the SIT, the intrinsic growth rate (r) depends on the intrinsic birth rate in daughters (b) but also the fertility function P. In the course of one generation, absent density-dependent effects,

$$\mathrm{e}^r \;=\; bP \;=\; b\left(\frac{\pi M}{\pi M + S}\right) \quad , \tag{A2}$$

with M as the number of fertile males (again, to be subscripted by genotype). Thus,

$$r = \ln(b) + \ln(\pi M) - \ln(\pi M + S) \quad . \tag{A3}$$

The model is intended to depict the evolution of female preference for fertile males over sterile males. We model a sexual haploid population. We will assume that the a allele exhibits no preference ($\pi = 1$) and that the A allele has preference for fertile males ($\pi > 1$). The basics of inheritance are such that an A mother produces all A sons when mating with an A sire (a fraction $M_A/(M_a + M_A)$ of all her matings), and produces half A sons when mating with an a sire (a fraction $M_a/(M_a + M_A)$ of all her matings). Thus a fraction

$$\frac{\frac{M_a}{2} + M_A}{M_a + M_A}$$

of all her matings result in A progeny. Likewise, half the offspring of a mothers are A sons when the sire is A.

With these preliminaries, differential equations for dynamics of females of each genotype become

$$\dot{F}_A = r_A F_A\left(1 - \frac{T}{K}\right)\frac{2M_A + M_a}{2M_T} + r_a F_a\left(1 - \frac{T}{K}\right)\frac{M_A}{2M_T} \; - \delta F_A \tag{A4}$$

$$\dot{F}_a = r_a F_a\left(1 - \frac{T}{K}\right)\frac{2M_a + M_A}{2M_T} + r_A F_A\left(1 - \frac{T}{K}\right)\frac{M_a}{2M_T} \; - \delta F_a \quad , \tag{A5}$$

where

$$M_j = F_j \;, \quad j \in \{a, A\} \;\; \text{(male numbers equal female numbers)} \tag{A6}$$

$$T = (F_A + M_A + F_a + M_a) \tag{A7}$$

$$r_a = \ln(b) + \ln(M_T) - \ln(M_T + S) \tag{A8}$$

$$r_A = \ln(b) + \ln(\pi M_T) - \ln(\pi M_T + S) \tag{A9}$$

$$M_T = M_a + M_A \quad . \tag{A10}$$

The intrinsic death rate (δ) merely prevents pathological outcomes in which all evolution stops when the population reaches carrying capacity. In models with periodic cessation of sterile male release, or with a constant-proportion release (see below), S becomes a function of time. In models with migration, the number of migrants is added to the respective equation.

Some noteworthy assumptions. We assume that each brood has b sons and b daughters. For simplicity, we equate the numbers of females and males in the population at all times. A more realistic approach would be to equate the births of sons and daughters and assign potentially different death rates to each. Doing so could change the speed of evolution and change the adult sex ratio, potentially impacting the effect of different values of S. This leads us to recognize a second set of key assumptions. Females (and thus fertile males) are born (b) and die (δ). In contrast, sterile males merely have a presence given by S. Implementations of the SIT would need to account not only for the abundance of sterile males relative to fertile males, but also the relative longevity of the two. If mating was seasonal, then abundances and longevity would matter only throughout the relevant season. But environments permitting ongoing matings and births would need to account for differential longevity of sterile and fertile males. As our interest is in identifying qualitative properties of evolution and dynamics, these simplifications are not limiting, but they would be in the design of field trials.

Comparing resistance evolution between proportional and constant release: discrete time

Consider a haploid population with discrete, nonoverlapping generations in which the number of female and male offspring of a given genotype are the same. Let N_a be the abundance of wild type females or males and N_A be the number of discrimination-bearing females or males. It is also useful to define

$$N = N_a + N_A$$

as the total number of males (females) and

$$p = \frac{N_A}{N}$$

as the frequency of discrimination-bearing males (females). The corresponding frequency of wild type males (females) is $q = 1 - p$.

If the preference of discriminating females is π and the per capita number of female (male) offspring per fertile mating is b, then the number of discriminating females (males) among the offspring are

$$\begin{aligned} N'_A &= b\left[\frac{\pi N}{\pi N + S}\left(p + \frac{q}{2}\right) N_A + \frac{N}{N+S}\left(\frac{p}{2}\right) N_a\right] \\ &= R_A \cdot N_A \end{aligned} \tag{A11}$$

where

$$R_A = \frac{b}{2}\left[\frac{\pi}{\pi + d}\cdot(1+p) + \frac{1}{1+d}\cdot q\right] \tag{A12}$$

is the finite growth rate of discriminators and

$$d = \frac{S}{N} \tag{A13}$$

is the ratio of released sterile to fertile males.

The corresponding dynamic for wild type females (males) is

$$\begin{aligned} N_a' &= b\left[\frac{N}{N+S}\left(q+\frac{p}{2}\right)N_a + \frac{\pi N}{\pi N+S}\left(\frac{q}{2}\right)N_A\right] \\ &= R_a \cdot N_a \end{aligned} \tag{A14}$$

where

$$R_a = \frac{b}{2}\left[\frac{1}{1+d}\cdot(1+q) + \frac{\pi}{\pi+d}\cdot p\right] \tag{A15}$$

is the wild type finite growth rate. In a population with only wild type, $q = 1$ and $p = 0$. A wild type population will decline as long as the release ratio $d > b - 1$.

With proportional release, d is a fixed parameter whereas with constant release, d is a variable that increases monotonically as population size declines. Note from (A12) that the growth rate of A decreases with d. If $\pi > 1$, the growth rate climbs with p, and since d is fixed for proportional release, the growth rate of A would accelerate as it spreads. (This may not ensure its persistence, however—see below.) With constant release, d is expected to increase at least initially which may counter any frequency-driven acceleration.

While $\pi > 1$ ensures the frequency of discriminators will increase, it does not ensure demographic persistence. A necessary condition for persistence is that the growth rate of A (see eq. A12) exceeds 1 when it is fixed ($p = 1$). This in turn defines the minimum preference required for persistence:

$$\pi_{\min} = \frac{d}{b-1} \tag{A16}$$

which is equivalent to the expression in the main text. Since $d > b - 1$ is required to eradicate the wild type, a preference that ensures long-term demographic persistence of discriminators ($\pi > \pi_{\min}$) also implies their evolutionary spread ($\pi > 1$) at the expense of the wild type. Since the growth rate of discriminators (A12) increases with p if d is fixed (i.e., proportional release) , it is theoretically possible that a mutant with $\pi > \pi_{\min}$ initially declines in abundance when rare.
